# An infantile case of hereditary folate malabsorption with sudden development of pulmonary hemorrhage: a case report

**DOI:** 10.1186/s13256-022-03448-x

**Published:** 2022-06-30

**Authors:** Yukari Sakurai, Naohisa Toriumi, Takeo Sarashina, Toru Ishioka, Marino Nagata, Hiroya Kobayashi, Hiroshi Azuma

**Affiliations:** 1 Department of Pediatrics, Asahikawa Medical University, 2-1-1-1, Midorigaoka-Higashi, Asahikawa, Hokkaido 078-8510 Japan; 2 Department of Pediatrics, Wakkanai City Hospital, 4-11-6, Chuou, Wakkanai, Hokkaido 097-8555 Japan; 3 Division of Immunopathology, Department of Pathology, Asahikawa Medical University, 2-1-1-1, Midorigaoka-Higashi, Asahikawa, Hokkaido 078-8510 Japan

**Keywords:** Case report, Hereditary folate malabsorption, Homocysteine, Megaloblastic anemia, *SLC46A1*

## Abstract

**Background:**

Hereditary folate malabsorption—a rare disorder caused by impairment of the folate transporter—can develop into severe folate deficiency manifesting as megaloblastic anemia and occasionally thrombocytopenia. Reportedly, megaloblastic anemia can manifest with hemorrhagic episodes, possibly due to ineffective platelet production and platelet dysfunction. However, life-threatening hemorrhage events in hereditary folate malabsorption have not been well investigated.

**Case presentation:**

A 3-month-old Japanese boy was transferred to our hospital due to thrombocytopenia and severe megaloblastic anemia. During a thorough examination of hematopoietic abnormalities, the patient suddenly went into cardiac arrest due to pulmonary hemorrhage. Although intravenous folate supplementation was started soon after the identification of folate deficiency, the patient died of circulatory defect and multiple organ failure. The cause of pulmonary hemorrhage, such as respiratory infection, could not be confirmed. Genetic investigation revealed a mutation in the *SLC46A1* gene to be the cause of the hereditary folate malabsorption.

**Conclusion:**

We report an infantile case of hereditary folate malabsorption that progressed to lethal pulmonary hemorrhage before folate deficiency was identified. Clinicians should consider that megaloblastic anemia could lead to severe bleeding without warning, and that nutrient supplementation should be initiated as soon as possible.

## Background

Megaloblastic anemia is characterized by ineffective hematopoiesis, which frequently manifests as decreased mature healthy red blood cells and unusually large, abnormal, and immature erythrocytes that fail to enter blood circulation due to their large size [1]. Leukopenia or thrombocytopenia is also present [1]. The abnormal erythrocytes called megaloblasts are fragile, resulting in intramedullary and extravascular hemolysis. The most frequent causes of megaloblastic anemia are deficiencies of either vitamin B12 or folate, both of which are cofactors required for DNA synthesis [2]. When DNA synthesis is impaired, the cell cycle cannot progress from the G2 growth stage to the mitosis (M) stage. This leads to cell growth without division, which manifests as unusually large, abnormal, and poorly developed erythrocytes (megaloblasts). In severe cases, thrombocytopenia, leukopenia, and hypersegmented neutrophils may be present, resulting from impaired nuclear differentiation [1].

Folate deficiency occurs due to poor dietary intake, exposure to antifolate drugs, and impairments in folate metabolism and transporters [2]. Hereditary folate malabsorption (HFM) is a rare autosomal recessive disorder caused by loss-of-function mutations in *SLC46A1*, the gene coding for the proton-coupled folate transporter (PCFT), which is an essential molecule for intestinal folate absorption and folate transport into the central nervous system [3]. Infants with this condition are normal at birth because of nourishment from the mother during fetal development. However, within a few months, they develop the symptoms of folate deficiency, such as anemia, immunoglobulin deficiency, infections, diarrhea, and later, neurological damage.

We present a case of HFM in a 3-month-old Japanese boy, which manifested with megaloblastic anemia and thrombocytopenia. Laboratory examination showed severe folate deficiency, and genetic analysis revealed the presence of a deep intronic mutation (c.1166-285 T>G of *PCFT-SLC46A1*), resulting in a 168-bp insertion in cDNA [4]. It was not until serum folate was found to be undetectable that the patient developed sudden pulmonary hemorrhage. Although life-threatening hemorrhage events in previously reported HFM cases have been rare, this case warns us to pay attention to the bleeding tendency of this disease.

## Case presentation

### Patient history

A 3-month-old Japanese baby was transferred to our institute due to severe anemia and thrombocytopenia. He was the first child of healthy, non-consanguineous parents with no family history of hematological or congenital disorders. During the first month of his life, he gained weight exclusively from breastfeeding; however, he gradually started vomiting and having diarrhea. Laboratory examinations revealed severe anemia (hemoglobin, 52 g/L) and thrombocytopenia (platelet count, 37 × 10^9^/L) with the presence of schistocytes (8% of red blood cells). Mean corpuscular volume, mean corpuscular hemoglobin, and mean corpuscular hemoglobin concentration were 84.4 fL, 30 pg, and 360 g/L, respectively. Reticulocyte elevation was inadequate (28‰ of red blood cells). Although lactate dehydrogenase was elevated to 1728 U/L, and total bilirubin and potassium were also elevated, direct and indirect Coombs tests were negative (Table 1).

**Table 1:** Results of laboratory examination at diagnosis

WBC	6.01 × 10^9^/L	PTINR	1.08	TP	53 g/L	Ferr	420 µg/L
Neut	0.84 × 10^9^/L	APTT	34.5 seconds	ALB	41 g/L	Fe	51 µmol/L
Lymp	4.99 × 10^9^/L	Fib	0.8 g/L	T-Bil	58 µmol/L	UIBC	4.7 µmol/L
		D-d	5340 µg/L	D-Bil	5.1 µmol/L	TIBC	56 µmol/L
RBC	1.73 × 10^12^/L	ATIII	76%	AST	70 U/L		
Hb	52 g/L			ALT	34 U/L	Folate	< 0.9 nmol/L
MCV	84.4 fL			LDH	1728 U/L	Vit.B12	31 pmol/L
MCH	30 pg	C3	0.6 g/L	BUN	3.9 mmol/L	Hp	< 0.10 g/L
MCHC	360 g/L	C4	0.2 g/L	Cre	22 µmol/L	Direct Coombs	(–)
Ht	0.146 fraction	CH50	5740 U/L	Na	138 mmol/L	Indirect Coombs	(–)
Plt	37 × 10^9^/L			K	5.0 mmol/L	O157 LPS Ab	(–)
Ret	4.8 × 10^9^/L	IgG	2.58 g/L	Cl	105 mmol/L	ADAMTS13	0.989 IU/mL
Schistocyte	138 × 10^9^/L	IgA	0.09 g/L	Ca	2.32 mmol/L		
		IgM	0.09 g/L	IP	1.32 mmol/L		
				CRP	< 1.0 mg/L		

*WBC* white blood cell, *Neut* neutrophil, *lymp* lymphocyte, *RBC* red blood cell, *Hb* hemoglobin, *MCV* mean corpuscular volume, *MCH* mean corpuscular hemoglobin, *MCHC* mean corpuscular hemoglobin concentration, *Ht* hematocrit, *Plt* platelet, *Ret* reticulocyte, *PTINR* international normalized ratio of prothrombin time, *APTT* activated partial thromboplastin time, *Fib* fibrinogen, *D-d* D-dimer, *ATIII* antithrombin, *C3* complement component 3, *C4* complement component 4, *CH50* 50% hemolytic unit of complement, *IgG* Immunoglobulin G, *IgA* Immunoglobulin A, *IgM* Immunoglobulin M, *TP* total protein, *ALB* albumin, *T-Bil* total bilirubin, *D-Bil* direct bilirubin, *AST* aspartate aminotransferase, *ALT* alanine aminotransferase, *LDH* lactate dehydrogenase, *BUN* blood urea nitrogen, *Cre* creatinine, *Na* sodium, *K* potassium, *Cl* chloride, *Ca* calcium, *IP* inorganic phosphorus, *CRP* C-reactive protein, *Ferr* ferritin, *Fe* ferrum, *UIBC* unsaturated iron binding capacity, *TIBC* total iron binding capacity, *Vit.B12* Vitamin B12, *Hp* haptoglobin, *O157 LPS Ab* O157 lipopolysaccharide antibody, *ADAMTS13* a disintegrin-like and metalloproteinase with thrombospondin type 1 motifs 13

**Fig. 1:**
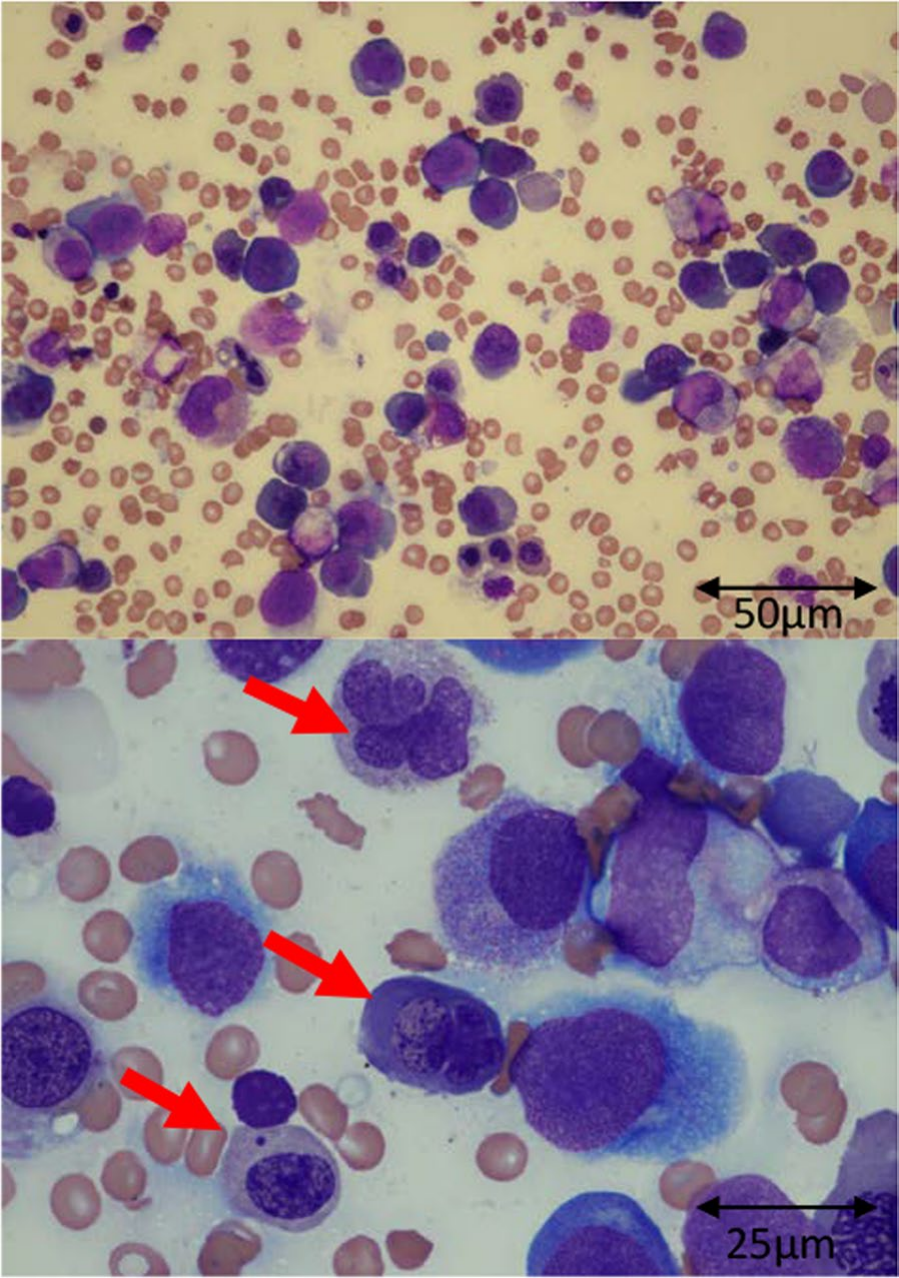
Bone marrow smear examination at diagnosis. May Grünwald–Giemsa staining, ×400 magnification (upper) and ×1000 magnification (lower). Hyperplasia of erythrocyte series was confirmed and the red arrows were showing megaloblastic change; the ratio of myeloid cells to erythroid cells was found to be markedly decreased to 0.36. The nucleated cell count was 105 × 10^9^/L. The number of megakaryocytes was reduced (0.016 × 10^9^/L) for hyperplastic bone marrow. No feature of malignancy was observed

**Fig. 2:**
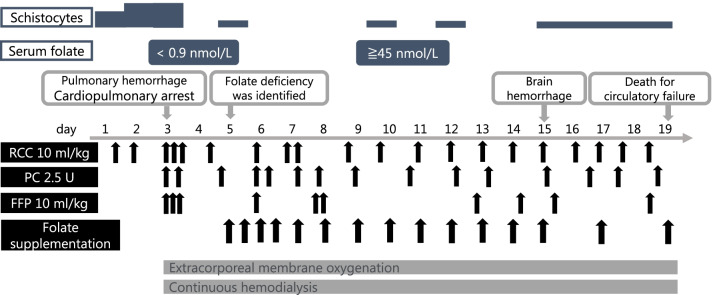
Clinical course of the patient. The patient developed pulmonary hemorrhage, resulting in cardiopulmonary arrest. Despite intensive care including extracorporeal membrane oxygenation, continuous hemodialysis, and folate supplementation, the patient did not recover from multiple organ dysfunction and hypoxic–ischemic encephalopathy

Bone marrow aspiration showed hyperplasia and megaloblastic change of erythroblasts, both of which are indicative of megaloblastic anemia. Dysplasia of granulocytes was also confirmed, and the percentage of megakaryocytes was decreased (Fig. 1). While further examinations were being performed, the patient suddenly developed pulmonary hemorrhage, resulting in cardiopulmonary arrest. Although the patient responded to cardiopulmonary resuscitation, he developed severe hypoxic–ischemic encephalopathy, respiratory insufficiency, and multiple organ dysfunction syndrome. Subsequently, he was started on intensive care including hemodialysis, plasma exchange, extracorporeal membrane oxygenation, and frequent blood transfusions. On the fifth day of hospitalization, serum folate level was found to be undetectable (normal, 11–34 nmol/L). This led us to confirm that folate deficiency was the cause of the megaloblastic anemia. Although intravenous folate supplementation (folinic acid, 6 mg/day) was initiated, the patient died of circulatory failure 19 days after hospitalization (Fig. 2). Examination of autopsy samples revealed that the megaloblastic change in his bone marrow had disappeared; however, the cause of pulmonary hemorrhage could not be confirmed. An amino acid analysis conducted before intravenous folate replenishment indicated high levels of serum homocysteine, serum cysteine, and urine cysteine, and a low level of serum methionine, which normalized after the administration of medication (Table 2). In Japan, all newborns are screened for metabolic disorders. On reanalysis of his neonatal blood sample, methionine, homocysteine, and cysteine levels were found to be normal (Table 2). Thus, the abnormal pattern of amino acids was consistent with HFM.

**Table 2:** Clinical variation of plasma amino acids associated with folate supplementation

	Normal value	At birth	Admission	After supplementation
Serum folate	11–34 (nmol/L)	N.A.	< 0.9	≥ 45
Serum methionine	18.9–40.5 (µmol/L)	24.014	8.8	29.7
Serum homocysteine	1.8–4.6 (µmol/L)	1.6	21.0	N.D.
Serum cysteine	13.7–28.3 (µmol/L)	22.9	32.4	15.9

*N.A.* not available, *N.D.* not detected

### Genetic analysis

This study was approved by the ethics committee of Asahikawa Medical University (no. 18231). Blood samples were collected from the patient and his parents after obtaining their informed consent. DNA was isolated using the Gentra Puregene blood kit (Qiagen, Hilden, Germany) in accordance with the manufacturer’s instructions. RNA was isolated from the father’s blood sample and used to synthesize complementary DNA (cDNA) by employing the SuperScript III first-strand synthesis system (Invitrogen, Carlsbad, CA, USA) for reverse-transcription polymerase chain reaction (RT-PCR). Direct sequence analyses targeting *SLC46A1* exons 1–5 and intron 3 were performed according to previous reports [3, 4]. The following primers were used in the process: exon1-forward, 5′-CGCCGGACATTTAAGGAG-3ʹ; exon1-reverse, 5′-ATGGTAGTGGCGGGTAACTG-3′; exon2(anterior half)-forward, 5′-AGGTTTAGGGCTCCAAAGGA-3′; exon2(anterior half)-reverse, 5′-TAAAGTGTGTGGGCTCAGGG-3′; exon2(posterior half)-forward, 5′-ATGCTGGCAAGCCTCCTC-3′; exon2(posterior half)-reverse, 5′-GAAATCCCTCAAAAATGCCA-3′; exon3-forward, 5′-TAGGAGCTCTGCTGCCTTTC-3′; exon3-reverse, 5′-TGTCTGGTTCCTCATGTCCA-3′; exon4-forward, 5′-GAATATGGCCCTTTCGGACT-3′; exon4-reverse, 5′-TGAACCCATGCTCATAATGG-3′; exon5-forward, 5′-AGGAGGAGGTTCAGGAGAG-3′; exon5-reverse, 5′-GCAAAGTGAACACCAAGCAA-3′; intron3-forward, 5′-AGCAGAAGGAGGAACAGGAA-3′; intron3-reverse, 5′-TCCACATATGACCTTACTGAATCC-3′.

**Fig. 3:**
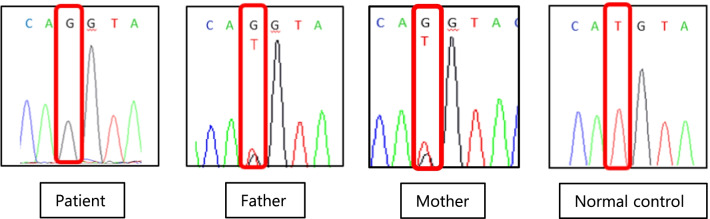
Electropherograms of c.1166-285T>G mutation for the patient and his parents. Sequence genomic deoxyribonucleic acid targeting c.1166-285T>G shows homozygosity of the mutation in the patient. His parents were heterozygous for the mutation. *A* adenine, *G* guanine, *T* thymine, *C* cytosine

Although no significant mutations were detected in the exon sequences of the patient, a homozygous mutation of c.1166-285 T>G was detected (Fig. 3) in intron 3. His parents were heterozygous for the mutation.

## Discussion and conclusions

Our patient was doing well, and folate deficiency was not suggested until he turned 1 month old. Folate and vitamin B12 play key roles in the methionine cycle to remethylate homocysteine to methionine, and the serum homocysteine level is elevated when there is a shortage of folate or vitamin B12 [2]. Based on this, it is possible that the patient was not deficient in folate at birth, because serum homocysteine at birth was not elevated (Table 2). In the subsequent few months, folate deficiency must have progressed due to impaired absorption through the intestine, resulting in the manifestation of megaloblastic anemia and thrombocytopenia. On admission to our hospital, the serum folate level was reduced to undetectable levels. On the contrary, the serum homocysteine level was found to be increased. The appearance of schistocytes in the peripheral blood may indicate the presence of dyserythropoiesis in case of folate deficiency [5].

Gene analysis revealed a c.1166-285 T>G mutation in intron 3 of *SLC46A1*. This replacement is reported to induce splicing changes and cause a 168-bp insertion between exons 3 and 4, leading to premature termination and development of HFM [4]. Both parents were heterozygous for this mutation.

Since Qiu *et al.* demonstrated the molecular basis for HFM [6], 36 cases of genetically confirmed HFM have been reported [7, 8]. HFM is generally diagnosed in early infancy; most patients develop recurrent infection, failure to thrive, or developmental delays from 2 months to 1 year of age. Megaloblastic anemia was present at onset in all cases, except in those who received early folate supplementation due to family history. Seizures tended to manifest in cases in which folate supplementation was delayed or started orally. Serum folate levels before supplementation were undetectable in more than half of cases.

Although only a few bleeding episodes have been reported in previous HFM cases, it should be noted that our patient exhibited a sudden and serious course of pulmonary hemorrhage. The bleeding occurred while preparing for a transfusion with a platelet count of 13 × 10^9^/L. Another patient with HFM developed alveolar hemorrhage concomitant with thrombocytopenia (18 × 10^9^/L) [4]. In addition, intracranial hemorrhage was reported in PCFT-deficient mice [9]. Judging from these facts, it is important to recognize bleeding tendency as one of the features of this disease.

Considering that prophylactic platelet transfusion is generally recommended at a platelet count equal to or less than 10 × 10^9^/µL in a variety of conditions involving thrombocytopenia [10], it cannot be simply assumed that a low platelet count was the cause of pulmonary hemorrhage. There might have been additional factors contributing to this event.

Hemorrhagic events such as retinal hemorrhages and purpuric spots have been reported in patients with megaloblastic anemia caused by vitamin B12 deficiency. Bleeding in the form of hematemesis, hemoptysis, and even life-threatening bleeding have also been reported [11]. Interestingly, Saloujin *et al.* reported that, in cases of megaloblastic anemia caused by vitamin B12 deficiency, the function of platelets is deteriorated [12]. Therefore, it is possible that platelet function is deteriorated in conditions of HFM. This might have been the factor that made the bleeding tendency much more severe than we speculated based on the platelet count, although we were unable to perform platelet function tests due to the severe clinical course.

Elevated serum homocysteine might cause endothelial dysfunction [13]. If hyperhomocysteinemia is severe (around 100 µmol/L), thromboembolism is often combined with endothelial damage, which may lead to thrombotic microangiopathy and diffuse alveolar hemorrhage, as previously reported in vitamin B12 disorder [14–16]. However, our case presented only mild elevation of homocysteine (21.0 µmol/L; normal range 1.8–4.6 µmol/L). This is within the recommended level (below 30 µmol/L) [17]. Therefore, a contribution of the elevated homocysteine to the bleeding tendency can be excluded.

We describe herein a pediatric case of megaloblastic anemia with HFM complicated by severe pulmonary hemorrhage. Thrombocytopenia and possible platelet dysfunction were considered to be the main causes. Life-threatening hemorrhage has not been well documented in HFM accompanied by megaloblastic anemia, possibly because of the rarity of HFM. However, it is undoubtedly an important complication of megaloblastic anemia. When an infant presents with megaloblastic anemia, folate or vitamin B12 deficiency should be assessed immediately, and supplementation of these elements should be initiated as soon as possible, even before the cause of megaloblastic anemia is identified.

## Data Availability

Not applicable.

## Abbreviations

HFM: Hereditary folate malabsorption
PCFT: Proton-coupled folate transporter
